# Applications of Aptamers in the Diagnosis and Treatment of Ovarian Cancer: Progress From 2016 to 2020

**DOI:** 10.3389/fgene.2021.683542

**Published:** 2021-09-13

**Authors:** Luoshan Ruan, Xin Li

**Affiliations:** Department of Gynecology, Renmin Hospital of Wuhan University, Wuhan, China

**Keywords:** aptamer, diagnosis, ovarian cancer, systematic evolution of ligands by exponential enrichment, treatment

## Abstract

Nucleic acid aptamers are short single-stranded DNA or RNA oligonucleotides selected from a random single-stranded nucleic acid library using systematic evolution of ligands by exponential enrichment technology. To allow them to bind to molecular targets with the same specificity and precision as that of antibodies, aptamers are folded into secondary or tertiary structures. However, compared to antibodies, aptamers are not immunogenic and are easier to synthesize. Furthermore, they are chemically modified, which protects them from degradation by nucleases. Hence, due to their stability and favorable targeting ability, aptamers are promising for the diagnosis and treatment of diseases. Ovarian cancer has the worst prognosis among all gynecological diseases and is usually diagnosed at the medium and advanced stages due to its nonspecific symptoms. Relapse is common, even if patients receive a standard therapeutic regimen including surgery and chemotherapy; simultaneously, drug resistance and adverse effects are reported in a several patients. Therefore, the safer and more efficient diagnostic and treatment method for ovarian cancer is imperative. Scientists have been trying to utilize aptamer technology for the early diagnosis and accurate treatment of ovarian cancer and some progress has been made in this field. This review discusses the screening of nucleic acid aptamers by targeting ovarian cancer cells and the application of aptamers in the diagnosis and treatment of ovarian cancer.

## Introduction

Nucleic acid aptamers, first reported by Ellington and Szostak (1990) and Tuerk and Gold (1990; hereinafter referred to as “aptamer”), are oligonucleotides screened by the systematic evolution of ligands by exponential enrichment (SELEX) technology from a random single-stranded nucleic acid (DNA or RNA) library. These oligonucleotides fold into secondary or tertiary structures, allowing them to bind to molecular targets with specificity and precision (Hoosen et al., 2018). To some degree, aptamers are like antibodies that are synthesized through chemical pathways; however, compared to antibodies, aptamers are more stable to variations in pH and temperatures, have easily modifiable chemical structures, and are not immunogenic. Aptamers also have lower molecular weights and exhibit better tissue penetration compared to antibodies. Moreover, aptamers can also bind to metal ions, peptides, drugs, proteins, cells, and viruses. Furthermore, aptamers can be synthesized in large quantities with high uniformity (Wu et al., 2015). Due to differences in the types and structures of bases, DNA and RNA aptamers have their respective advantages and disadvantages. RNA aptamers can fold into more three-dimensional structures because of the presence of 2′-OH groups and the non-Watson-Crick base pairing, resulting to more simplified screening (Sun et al., 2014). Meanwhile, DNA aptamers are naturally resistant to 2′-endonucleases because they lack 2′-OH and are more stable *in vivo* (Sun et al., 2014).

Due to their favorable targeting ability, aptamers have extensive applications in cancer detection and treatment. Ovarian cancer is the most fatal gynecological malignancy, leading to more deaths than that of all other gynecological cancers combined, thereby being a significant threat to women’s health. Worldwide, approximately 240,000 new cases are reported annually, about two-thirds of which are fatal (Henri et al., 2018). Symptoms of ovarian cancer, such as abdominal distension, indigestion, and nausea, are nonspecific; therefore, ovarian cancer is often misdiagnosed as gastrointestinal ailments and is difficult to diagnose at an early stage (Liu et al., 2019), with approximately 75% patients diagnosed at advanced FIGO stages (Hoosen et al., 2018). The survival rate for women diagnosed with ovarian cancer at advanced stages is low, and the five-year survival rate of patients at stage III–IV is only 17–39% (Liu et al., 2019). Even if treated with standard surgery and chemotherapy, only 20–25% patients are cured effectively; recurrence, unbearable adverse effects, and drug resistance are reasons for unsuccessful treatment (Hoosen et al., 2018). Therefore, early diagnosis and accurate treatment are critical for curing ovarian cancer. In recent years, scientists have begun evaluating aptamers for the diagnosis and treatment of ovarian cancer. This article discusses the screening aptamers used in targeting ovarian cancer and applications of aptamers in diagnosis and treatment of ovarian cancer using articles published on PubMed in the last 3 years.

## Screening for Aptamers Targeting Ovarian Cancer

SELEX technology selects aptamers that specifically bind to molecular targets through a process of reduplicative selection and amplification. The process involves five steps: incubation, separation, elution, amplification, and conditioning. There are 10^13^–10^16^ random sequences in a DNA or RNA library (Wu et al., 2015). Hence, to ensure the accurate binding of aptamers to their molecular targets, a negative selection using non-target molecules can be performed to remove unwanted aptamers that bind to non-target molecules (Zhu and Chen, 2018). Ideal aptamers are obtained after 8–15 rounds of selection and elution (Wu et al., 2015). DNA aptamers are amplified by polymerase chain reaction, whereas RNA aptamers are amplified by reverse transcription-polymerase chain reaction (Xiang et al., 2015). The traditional SELEX process is complex and time-consuming; therefore, affinity chromatography, magnetic separation technology, kinetic capillary electrophoresis, and microfluidic strategies are used to shorten the time of separation (Wu et al., 2015). Table 1 lists the new aptamers screened in the last 3 years.

**Table 1:** Newly screened aptamers targeting cancer-specific molecules and their characteristics.

Aptamers	Dissociation constant (*K_d_*)	Application	Target	References
CA125_1 (DNA) CA125_12 (DNA)	270 ± 109 U/ml, 80 ± 38 U/ml 118 ± 123 U/ml, 131 ± 93 U/ml	Not mentioned	CA125	Scoville et al., 2017
2.26	166 nm	Not mentioned	CA125	Tripathi et al., 2020
Heraptamer1 (DNA) Heraptamer2 (DNA)	5.1 ± 5.3 nm 23.7 ± 11.2 nm	SKOV3 cells SKOV3 cells	HER2, ECD	Zhu et al., 2017
Apt928 (DNA)	66 nm/L	SKOV3 cells	CD70	Bayat et al., 2019
HF3-58 (DNA) HA5-68 (DNA)	0.30 ± 0.24 nmol/L 4.5 ± 1.6 nmol/L	A2780T cells A2780T cells	A kind of glycoproteins on the cellular membrane	He et al., 2019
V5	Not mentioned	Patients’ tissue sections	Vimentin	Wang et al., 2016
Tx-01	53.8 ± 14.9 nm	Patients’ tissue sections	GPM6a, BRCA2	Hung et al., 2018
cTX-24 (DNA) cTX-36 (DNA) cTX-45 (DNA)	1,348 ± 518.1 nm 129.2 ± 24.81 nm 178.0 ± 43.5 nm	Patients’ tissue sections	Not mentioned	Liu et al., 2019

CA125 is the gold standard biomarker for ovarian cancer, and the serum CA125 levels are usually used to monitor the patients with ovarian cancer. Scoville et al. (2017) screened DNA aptamers with affinity for CA125 using the One-Pot SELEX approach, in which selection and amplification occurred in one container, thereby minimizing transfer steps that contribute to loss of product, and evaluated the CA125 binding affinities of candidate aptamers using fluorescence anisotropy and affinity probe capillary electrophoresis. Eventually, two aptamers, named CA125_1 and CA125_12, were selected. Tripathi et al. (2020) used membrane-SELEX technology, combined with bioinformatics, to screen for aptamers that bind to CA125. Subsequently, they identified aptamer 2.26, evaluated its dissociation constant using three separate assays, and verified its stability in serum and high salt solutions. From the studies by Scoville et al. (2017) and Tripathi et al. (2020), we can draw the conclusion that researchers should evaluate aptamer dissociation constants with several types of assays to ensure accuracy and select the best aptamers. Moreover, even if the target protein is same, different aptamers may recognize different domains; hence, selecting multiple aptamers to the same target protein may not be a redundant exercise. Additional, researchers should consider flexible utilization of tools from other fields of study.

HER2 is an epidermal growth factor receptor family protein overexpressed in approximately 30% of cases of ovarian cancer, breast cancer, and other types of cancers, and its overexpression is related to poor prognosis, tumor metastasis and relapse, chemotherapeutic resistance, and low survival rate. HER2 comprises an extracellular domain (ECD) of approximately 630 amino acids, a transmembrane domain, and a cytoplasmic domain of tyrosine kinase. Zhu et al. (2017) screened DNA aptamers with affinity for the HER2 protein by successively using HER2 ECD and live HER2-overexpressing SKOV3 cells. Subsequently, these aptamers were radiolabeled with isotope F^18^ to verify the binding of aptamers and HER2-overexpressing SKOV3 tumor model with positron emission tomography (PET), using HER2-negative MD-MBA-231 cells as the control. Finally, two aptamers, Heraptamer1 and Heraptamer2, were selected.

CD70 is a type II transmembrane protein that is part of the tumor necrosis factor superfamily. Its overexpression is related to a series of tumors including ovarian cancer. Bayat et al. (2019) screened DNA aptamers with affinity for histidine-labeled recombinant CD70 protein and CD70-overexpressing SKOV3 cells and identified that Apt928 could obstruct the combination of CD70 and CD27. The detection limit in SKOV3 cells was determined by labeling Apt928 with ATTO 674 N. Although the target proteins mentioned here are not recommended ovarian cancer biomarkers, there are different types of ovarian cancers with different proteins expressed during tumorigenesis. As these proteins may play different functional roles, seeking new ovarian cancer biomarkers and screening corresponding aptamers for tools to improve diagnosis and treatment of ovarian cancer are necessary.

Due to posttranslational modifications, especially in the case of highly glycosylated proteins, purified proteins, or peptides often cannot fold into the correct three-dimensional structures that are formed under physiological conditions. As a result, aptamers could not bind to target proteins; therefore, scientists invented the Cell-SELEX technology-based cells (Sun et al., 2014). Cell-SELEX technology screens aptamers for any cells without the prior knowledge of their targets; moreover, it can also be used to discover unknown biomarkers (Zhu and Chen, 2018). For example, He et al. (2019) screened DNA aptamers with affinity for paclitaxel (PTX)-resistant ovarian cancer cell line A2780T, while using the wild-type A2780 cells (sensitive) as a negative control, and selected two aptamers, named HF3 and HA5. A strategy involving truncation and mutation was used to optimize these two aptamers and infer the key aptamer structures that bind to drug-resistant cells. This resulted to the identification of two aptamers, named HF3-58 and HA5-68, that were verified to bind to a glycoprotein on the cellular membrane.

Besides purified proteins and cells, ovarian cancer tissue sections can also be used as targets to select aptamers. Wang et al. (2016) selected aptamers with affinity for ovarian tumors using a DNA library modified with thiophosphate substitutions of the phosphate ester backbone at selected 5′ dA positions. An image-directed laser microdissection technology based on morphological assessment was used to dissect thioaptamer binding regions, and mass spectrometry was used to identify the molecular target. Eventually, vimentin was determined as the target protein of aptamer V5. Wang et al. (2016) called this method as Morph-X-Select.

Recently, integrated microfluidic systems have witnessed rapid development. Hung et al. (2018) developed a microfluidic system that could automate tissue-SELEX and phage display technology using ovarian cancer tissue sections and identified GPM6a and BRCA2 as the target molecules of aptamer Tx-01 using liquid chromatography/mass spectrometry analysis. Moreover, Liu et al. (2019) designed a microfluidic chip that could automate tissue-SELEX in six rounds within 15 h, screened three DNA aptamers, namely, cTX-24, cTX-36, and cTX-45, and measured their dissociation constant by flow cytometry.

Although the nature of the samples is different, Cell-SELEX and tissue-SELEX can both be used to screen for aptamers without specifying target proteins *a priori*. These two SELEX approaches offer convenience; they reduce the time required for seeking specific target molecules while allowing scientists to discover more proteins related to ovarian cancer, using multiple ovarian cancer cells or clinical specimens.

## Applications of Aptamers in Ovarian Cancer Diagnosis

### Detection Probe

**Figure 1:**
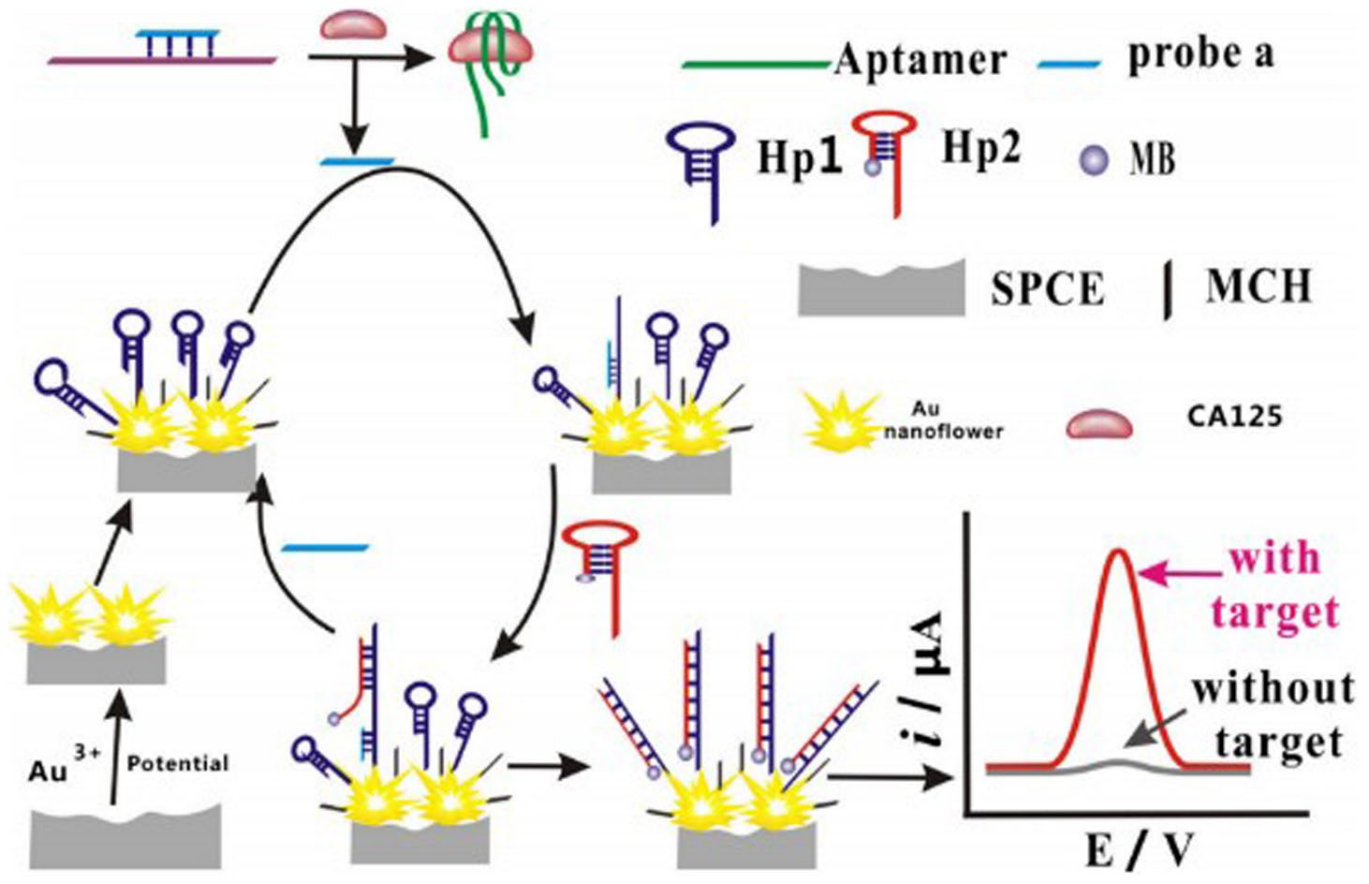
Diagram depicting the strand displacement amplification reaction.

Many researchers have designed molecular probes using aptamers targeting CA125, matching different nanomaterials labeled with fluorescent or chemical groups to generate fluorescent or electrochemical signals, respectively. Nie et al. (2018) immobilized H1 DNA strands on gold nanoparticles, followed by performing a hybridization chain reaction to induce a dendrimer-like chain reaction, resulting in increased generation of electrochemical signal when aptamers bind to CA125. This enhanced the sensitivity of the assay and lowered the limit of detection. Chen et al. (2019) electrodeposited flower-like gold nanostructures on a screen-printed carbon electrode to increase the sensor surface, allowing the assembly of more Hp1 nucleic acid strands. Hence, strand displacement amplification enhanced the electrochemical signal triggered by CA125 (Figure 1). Hamd-Ghadareh et al. (2017) used carbon dots and aptamers as detection probes so that when aptamers bind to CA125, the fluorescence signal generated by the carbon dots are quenched by PAMAM-dendrimers/Au nanoparticles (AuNPs) and CA125-antibodies. While the PAMAM-dendrimers could not enhance the fluorescence quenching ability of AuNPs, attaching more antibodies increased the detection ability of the probes. To amplify the electrochemical signal and attach more aptamers, Farzin et al. (2019) immobilized amidoxime modified with Ag nanoparticle polyacrylonitrile nanofibers by electrospinning on an electrode. When aptamers are combined with CA125, the methylene blue-labeled complementary DNA strands separate from the aptamers, generating an electrochemical signal. Quantum dots (QDs) are semiconductor structures that fetter excitons in three-dimensional directions. Jin et al. (2017) modified the surface of Ag_2_S QDs with polyethyleneimine and used Ag_2_S QDs/aptamers/5-Fu as detection probes. When CA125 combined with the aptamer/5-Fu complex, the complex separated from the surface of QDs, inducing a “turned-on” phenomenon attributed to the recovery of near-infrared photoluminescence. Hu et al. (2020) grew AuNPs on a gallium nitride semiconductor *via* the Au-S bonds to fabricate a Schottky junction, then connected the aptamers targeting CA125 with the AuNPs *via* Au-S bonds. When the aptamers bound CA125, the photoelectron transfer process of the system was stopped, causing the photocurrent of the system to decrease.

In a different approach, Mansouri Majd and Salimi (2018) chose to employ a field effect transistor as a sensor, wrapped aptamers around carbonylated multiwalled carbon nanotubes, and immobilized these complexes on the poly-methyl methacrylate platform deposited with few layers of reduced graphene oxide. When aptamers bind to varying concentrations of CA125, this aptasensor generates different levels of current, while detecting the CA125 concentration. In all the above-mentioned cases, researchers designed detection probes with aptamers targeting CA125; however, the linear ranges and detection limits were different because they used different nanomaterials and the process to generate fluorescent or electrochemical signals was different. Although a lower detection limit demonstrated better sensitivity, the extremely low CA125 concentrations had no clinical significance. Nevertheless, the linear ranges of the detection probes should be of high value to ovarian cancer patients.

Carcinoembryonic antigen (CEA) is also a biomarker of ovarian cancer. Xu et al. (2020) designed a dual-fluorescence probe that could detect CEA and CA125 simultaneously. When CEA or CA125 bound to the corresponding aptamer, the DNA-silver nanocluster-aptamer complexes were dragged from AuNPs, which caused AuNPs to aggregate and a fluorescent signal to be generated. Aside from CA125, other molecules can also be used to design the detection probes. Mucin 1 (MUC-1) is a glycoprotein overexpressed on surface of most of malignant epithelial cells, such as those from colorectal, lung, prostate, pancreatic, ovarian, and bladder cancer, but not in normal human epithelial cells, making it an effective early diagnostic indicator (Cao et al., 2017). Cao et al. (2017) used an Immuno-Loop-Mediated Isothermal Amplification technology based on MUC-1 specific aptamers for the quantitative detection of MUC-1, which could detect low-abundance proteins with high sensitivity, at a detection limit of 120 MUC-1 molecules, without the need for fluorescent molecules. In summary, compared to current clinical detection approaches, detection probes designed based on aptamers do not require expensive monoclonal antibodies and offer a lower detection limit. Table 2 lists the linear range and detection limit of probe-based aptamers for detecting cancer biomarkers.

**Table 2:** Characteristics of signal probes-based aptamers.

Signal probes	Linear range	Detection limit	Target	References
Dendrimer formed by hybridized chain immobilized on AuNPs +aptamers	100 μU/ml–10 U/ml	50 μU/ml	CA125	Chen et al., 2019
Aptamers+carbon dots (CDs)	1.0 fg/ml–1.0 ng/ml	0.5 fg/ml	CA125	Hamd-Ghadareh et al., 2017
Aptamers+methylene blue-labeled complementary DNA strands	0.01–350 U/ml	0.0042 U/ml	CA125	Farzin et al., 2019
Aptamers/5-Fu /Ag_2_S quantum dots complex	0.1–10^6^ ng/ml	0.07 ng/ml	CA125	Jin et al., 2017
AuNPs+GaN Schottky junction+aptamers	1–100 U/ml	0.3 U/ml	CA125	Hu et al., 2020
DNA silver nanoclusters+ aptamers+AuNPs	0.01–0.9 ng/ml 0.01–2 U/ml	7.5 pg/ml 0.015 U/ml	CEACA125	Xu et al., 2020
Multiwalled carbon nanotubes (MWCNTs)/aptamers complex	1.0 × 10^−9^–1.0 U/ml	5.0 × 10^−10^ U/ml	CA125	Mansouri Majd and Salimi, 2018
MUC-1 aptamers	1.0 amol–1.0 pmol	120 MUC-1 molecules	MUC-1	Cao et al., 2017

Apart from detecting cancer biomarkers, aptamers can also be used in other aspects of cancer diagnosis. Bayat et al. (2019) used the newly screened aptamer Apt928 to detect the number of cancer cells; the detection limit of their aptasensor was 14 cells/ml, and the detection range was 10 to 10^5^ cells/100 μl. Tsai et al. (2017) designed an integrated microfluidic system using the CX-BG1-10-A aptamer for the detection of ovarian cancer-derived BG-1 cells, and the detection steps included RBC lysis, WBC depletion, and circulating tumor cell isolation, which made it more sensitive compared to antibody-based detection systems; this microfluidic system completed the entire process within an hour without human intervention and exhibited a high capture rate and a low false positive rate, thereby saving time. Coincidently, Wu et al. (2021) also designed a microfluidic system to capture cancer cells with aptamers that accordingly targeted EpCAM and N-cadherin, respectively. Growing experimental evidence indicates that the epithelial-to-mesenchymal transition participates in the progression of ovarian cancer. EpCAM is an epithelial marker and N-cadherin is a mesenchymal marker; therefore, employing these two aptamers simultaneously could increase the rate of capture.

In general, employing aptamers to design detection probes have wide application prospects, and unprecedented technological developments lay the foundation for using aptamers in the diagnosis of ovarian cancer. Employing multiple aptamers that target different cancer biomarkers could increase the positive rate of cancer cell, or cancer-related protein, detection. Future studies should consider means to detect ovarian cancer at earlier stages, using a combination of several cancer biomarkers. As demonstrated here, integrated microfluidic systems are a preferred platform for such research. This detection system on a chip has a referential significance for inventing effective and noninvasive cancer diagnostic methods.

### Other Applications

In addition to designing detection probes or capturing cancer cells, aptamers have other applications in ovarian cancer diagnosis. Huang et al. (2020) designed a microfluidic system for rapid, automatic, and high-throughput staining of tissue samples of ovarian cancer, using aptamer Tx-01, to perform fluorescent and immunohistochemistry staining that saved time and reagents, while making ovarian cancer diagnosis easy. In addition to staining, aptamers modified with fluorescent groups can be used for *in vivo* imaging. Zhu et al. (2017) labeled the screened aptamers Heraptamer 1 and Heraptamer 2 with isotope ^18^F, followed by PET imaging in a mouse model of ovarian cancer induced by SKOV3 cells, which exhibited high signal-to-background ratios within a short time and prevented aptamer degradation by nucleases. Li et al. (2018) injected Cy5-labeled R13 aptamer into nude mice and performed fluorescent imaging for tumor and organs half an hour later. A weak fluorescent signal was obtained in the first 1.5 h, which peaked at 3.5 h, and lasted for 9.5 h. Zhu et al. (2017) and Li et al. (2018) demonstrated the feasibility of using aptamers for *in vivo* imaging and suggested more approaches for ovarian cancer diagnosis.

## Applications of Aptamers in Ovarian Cancer Treatment

### Free Aptamers as Therapeutics

When aptamers bind to cell-surface molecules usually involved in cellular metabolic pathways, they are internalized into the cell with these molecules and bind to the functional sites of the cell-surface molecules, exerting an agonist, or antagonist function, depending on the cellular localization and molecular function of the target molecules (Wan et al., 2019). Therefore, it is feasible to use free aptamers as therapeutics. Macugen, which treats age-related macular degeneration by targeting VEGF (Wu et al., 2015), was the first aptamer approved to enter the market. Some aptamers have also been used in clinical trials. As for ovarian cancer, scientists are trying to use aptamers as target drugs. AXL receptor tyrosine kinase (AXL-RTX) is a key target for the treatment of metastatic and advanced-stage ovarian cancer and its expression significantly reduces the survival rate of patients with ovarian cancer. Using *in vitro* experiments, Kanlikilicer et al. (2017) demonstrated that the AXL-APTAMER targeting AXL-RTX could inhibit the activity of AXL-RTX and could also inhibit the growth, proliferation, migration, and invasive ability of AXL-RTX overexpressing ovarian cancer cells SKOV3-IP1 and HeyA8. Combined use of AXL-APTAMER and PTX effectively inhibited the tumor growth. Amero et al. (2021) selected phospho-AXL (p-AXL) as a target. They reversed the RNA aptamer into DNA aptamer and modified the aptamer backbone with thiophosphate and dithiophosphate to covalently link the 5′-end with polyethylene glycol in order to enhance stability and pharmacokinetic properties and prevent nuclease hydrolysis and renal clearance. Then, they screened two aptamers named GLB-G25 and GLB-A04, among 17 candidate aptamers, that mostly decreased the expression of p-AXL and suppressed ovarian cancer cell invasion and migration. Zheng et al. (2017) developed a bispecific aptamer capable of blocking CD44 and EpCAM simultaneously to inhibit cell growth and induce apoptosis in intraperitoneal ovarian cancer cells. CD44 signals to facilitate the growth, survival, and metastasis of ovarian cancer cells and can also serve as a receptor to mediate the attachment of ovarian cancer cells into the peritoneal mesothelial cells. Besides, CD44 is also related to ovarian cancer chemoresistance, and specimens obtained from patients with ovarian cancer have shown that CD44 expression is associated with high-grade and advanced-stage ovarian carcinoma. Meanwhile, EpCAM expression in the peritoneal cavity appears to be tumor-specific because mesothelial cells in the abdominal cavity do not express EpCAM, which inhibits cell-cell adhesion and epithelial-mesenchymal transition that are related to the ovarian cancer progression. Both CD44 and EpCAM are key markers of ovarian cancer stem cells and are responsible for peritoneal metastatic dissemination and chemoresistance. Therefore, Zheng et al. (2017) linked two aptamers that specifically bind to CD44 and EpCAM *via* a double-stranded RNA adaptor, which not only increased their half-life in circulation, but also reduced renal filtration compared to use of a single aptamer. At the same time, results of cell-based studies and animal trials have provided support that this fused aptamer inhibits ovarian cancer cell growth and suppresses intraperitoneal tumor progression more efficiently than CD44 and EpCAM aptamers used alone or in combination. In the view of above-mentioned experiments, we support chemically modifying aptamers to increase the molecular weight and nuclease resistance when developing free aptamers as potential ovarian cancer drugs. We also recommend identifying more apoptosis targets to exploit new aptamers.

### Aptamers as Chemotherapeutic Drug Delivery Systems

Standard chemotherapy regimens for ovarian cancer include the combination of PTX and platinum, and the use of liposomal doxorubicin and ifosfamide. Although the formulations of these chemotherapeutic drugs have improved, their nonspecific cytotoxicity makes the adverse effects unbearable (Henri et al., 2018). In addition, drug resistance is one of the causes of chemotherapy failure. To overcome this, the need for active targeting has been raised in the medical field. Active targeting guides therapeutic agents by targeting ligands to tumor cells and promoting their cellular entry *via* receptor-mediated endocytosis (Xiang et al., 2015). Therefore, many scientists are trying to take advantage of active targeting to cure patients with ovarian cancer. The water solubility of PTX is poor, and its side effects are severe, so Li et al. (2017) linked aptamer NucA targeting nucleolin and PTX *via* a cathepsin B sensitive valine-citrulline dipeptide bond. Once connected to NucA, the activity of PTX became very low or null. The dipeptide bond was hydrolyzed by cathepsin B only when NucA-PTX was delivered into the cell. Docetaxel (DTX) is a kind of PTX drug than can cause hypersensitivity reactions, fluid retention, neuro and musculoskeletal toxicity, and neutropenia due to its active pharmaceutical ingredient and the components of the solvent system. To address this, Ghassami et al. (2018) fabricated polymeric nanoparticles Ecoflex with an electro-spraying technique and loaded it with DTX and an aptamer targeting HER2 to increase the targetability of DTX while decreasing its adverse effects.

Doxorubicin cytotoxicity is caused by its intercalation into the nucleic acid structure at the paired CG or GC sites. Taking advantage of its propensity for intercalation, doxorubicin can be noncovalently conjugated to oligonucleotide aptamers containing CG/GC sequences through a simple incubation step (Sun et al., 2014). Pi et al. (2017) used a three-way junction motif from phi29 packaging RNA as the supporting structure; aptamer Endo28 targeting annexin A2 hybridized with this RNA motif and loaded doxorubicin with a GC-rich sequence to increase the target ability of doxorubicin for ovarian cancer.

The above-mentioned groups all linked aptamers with chemotherapy drugs, either directly or indirectly, to achieve an active targeting treatment. This not only decreased the side effects of the chemotherapy drugs but also increased the killing effect on the tumor cells, indicating that aptamers show great promise as non-toxic drug delivery molecules. Apart from chemotherapeutic drugs, researchers are also trying to use RNAs to knockdown genes and make use of aptamers to guide the therapeutic modality. MicroRNAs are noncoding endogenous RNA, consisting of 18–24 nucleotides, and their combination with the 3′-untranslated region (UTR) of a target mRNA may inhibit expression of target proteins or signaling pathways (Jiang et al., 2020). MicroRNA29b (miR-29b) plays a role in killing tumor cells by influencing cell proliferation, differentiation, and apoptosis and can target genes related to DNA methylation, cell cycle proteins, and cell apoptosis. Jiang et al. (2020) loaded miR-29b with cationic liposomes and connected liposomes with aptamer AS1411 specific for nucleolin. Liposomes increased the loading rate of drugs and circulation half-life and the positive charge made it easy to combine with cells that had a negative charge, demonstrating that this aptamer/liposome/miR-29b could kill ovarian cancer cells A2780. Vandghanooni et al. (2020) loaded cisplatin and locked nucleic acid (LNA) anti-miR-214 with polymeric nanoparticles and connected aptamer AS1411 to the nanoparticles. MicroRNA214 (miR-214) is overexpressed in cisplatin-resistant ovarian cancer cells and downregulates the expression of PTEN proteins, while activating the PI3K/Akt pathway by modulating the 3′-UTR of the PTEN gene, which is beneficial for cancer cell survival; therefore, inhibition of miR-214 may improve the sensitivity of tumors to chemotherapeutic drugs. Vandghanooni et al. (2018) further proved that their drug-loading complex could downregulate miR-214 and recovered the sensitivity of cisplatin-resistant ovarian cancer cells A2780 for cisplatin. MicroRNA21 (miR-21) plays key roles in tumor drug resistance, progression, and metastasis. The upregulation of miR-21 was directly related to the chemoresistance of ovarian cancer *via* promoting cancer cell survival (Vandghanooni et al., 2018). Vandghanooni et al. (2018) designed PEGylated poly (lactic-co-glycolic acid) polymeric nanoparticles loaded with antisense oligonucleotides of miR-21 and aptamer AS1411, which first decreased the level of miR-21 in cisplatin-resistant A2780 cells, reversed the drug resistance of cancer cells, and then delivered polymeric nanoparticles loaded with cisplatin and AS1411 to cells, thereby increasing the killing effect of cisplatin on cancer cells. Small interfering RNAs (siRNAs) are double-stranded RNAs that usually consist of 20–25 base pairs and can silence one sequence of mRNA. Chen et al. (2017) loaded aptamers targeting VEGF and Notch3 siRNA chimera with Au-Fe_3_O_4_ nanoparticles, and compared to single siRNA or siRNA-liposome, this complex downregulated the Notch3 gene more effectively and reversed the drug resistance of multi-drug resistant ovarian cancer cells. *PRKCI* is the gene for protein kinase Cι (PKCι). Rehmani et al. (2020) first identified that PRKCI is usually upregulated in high-grade serous ovarian cancer and relapsed patients; additionally, MTT assays showed that small-molecule PKCι inhibitors could not effectively induce apoptosis in PRKCI-amplified ovarian cancer without toxicity to normal ovarian and fallopian tube epithelial cells. The authors designed an EpCAM aptamer-PKCι siRNA chimera (AsiC) and verified that it could induce apoptosis in PRKCI-amplified ovarian cancer cells while suppressing the progression of intraperitoneal ovarian cancer in a mouse xenograft model.

## Conclusion

The prospects of using aptamers for tumor diagnosis and treatment are immense. However, due to the complex aptamer screening process, efforts must be directed toward the invention of simple, rapid, and automated instruments for screening aptamers. At the same time, other tumor markers of ovarian cancer need to be identified, and aptamer-based probes should be designed to detect the low-abundance proteins or for *in vivo* imaging, so that ovarian cancer can be diagnosed at an early stage. In addition, due to the low molecular weight of aptamers, appropriate materials should be explored to decrease their clearance rate or combined with anti-tumor drugs to improve their therapeutic effects, while decreasing the adverse effects. Besides, valuable gene sites should be screened, and nanomaterials loaded with aptamers and miRNAs, LNAs, and siRNAs should be explored to upregulate or downregulate the expression of target genes. Ideal aptamer/drug systems specific for tumor tissues *in vivo*, with low distribution rate in other tissues, remain to be designed. Recently, DNA self-assembly technology has caught the attention of scientists. As this technology enables folding the DNA in almost any shape with nanoscale precision, designing drug delivery systems and detection probes based on DNA self-assembly to target ovarian cancer may not remain a distant reality. Thus, researchers in the medical field should take advantage of aptamers to widen the diagnostic and therapeutic approaches for ovarian cancer.

## Author Contributions

XL proposed the conception, conducted the study, and revised all sections of the manuscript. LR organized the material and wrote the first draft of the manuscript. All authors contributed to manuscript revision, read, and approved the submitted version.

## Conflict of Interest

The authors declare that the research was conducted in the absence of any commercial or financial relationships that could be construed as a potential conflict of interest.

## Publisher’s Note

All claims expressed in this article are solely those of the authors and do not necessarily represent those of their affiliated organizations, or those of the publisher, the editors and the reviewers. Any product that may be evaluated in this article, or claim that may be made by its manufacturer, is not guaranteed or endorsed by the publisher.
